# Adsorption of Remazol Brilliant Violet-5R Textile Dye from Aqueous Solutions by Using Eggshell Waste Biosorbent

**DOI:** 10.1038/s41598-020-65334-0

**Published:** 2020-05-20

**Authors:** Eszter Rápó, László Előd Aradi, Ábel Szabó, Katalin Posta, Robert Szép, Szende Tonk

**Affiliations:** 1 Environmental Science Department, Sapientia Hungarian University of Transylvania, Calea Turzii no. 4, 400193 Cluj-Napoca, RO Romania; 2 Institute of Genetics, Microbiology and Biotechnology, Szent István University Páter Károly no. 1, 2100 Gödöllő, HU Hungary; 3 Lithosphere Fluid Research Lab, Eötvös Loránd University, Pázmány Péter sétány no. 1/c, H-1117 Budapest, HU Hungary; 4 Faculty of Technical and Social Sciences, Sapientia Hungarian University of Transylvania, Piaţa Libertăţii no. 1, 530104 Miercurea-Ciuc, RO Romania; 5 Institute for Research and Development for Hunting and Mountain Resources, Progresului no. 35B, 530240 Miercurea-Ciuc, RO Romania

**Keywords:** Pollution remediation, Environmental impact

## Abstract

Based on the well-known excellent adsorbent ability of chicken eggshells, the adsorptive capacity and mechanism of Remazol Brilliant Violet-5R (RBV-5R) dye by eggshell was investigated. Exploiting the high surface-area-to-volume ratio and porous structure of this natural adsorbent, the developed procedure showed to be useful for the efficient adsorption of RBV-5R dye from contaminated water. The protocol was thoroughly optimized by investigating the effect of the dye concentration, biomass-contaminated water ratio, particle size of the adsorbent, pH and temperature, as they are key factors in the efficiency of the dye removal process. The eggshell material was characterized by different types of microscopy techniques (stereo, polarization, SEM) as well as elemental analysis (element distribution mapping, EDX), Raman spectroscopy and BET-surface density measurements. EDX, FTIR and Raman spectroscopy proved the presence of the adsorbed dye on the surface of the biomaterial. It was shown that under optimal conditions, the environmentally friendly and inexpensive eggshell could be a reliable adsorbent for Remazol dye removal from wastewater.

## Introduction

One of today’s global challenges is to supply healthy, pure and consumable water to safeguard public health since a growing variety of toxic organic and inorganic pollutants are occurring in water bodies^1^. According to the 2015 World Water Development Report, the demand for water around the world will increase by 55% over the next 15 years, indicating that the Earth’s current water supplies can cover only 60% of our future needs by 2030^2^. With rapid industrial development, the overexploitation of natural resources, industrial and natural disasters, the number of polluted water sites contaminated with heavy metals^3–6^, radioactive elements^7,8^, nitrite^9,10^, phosphate^11,12^, pesticides^13^, dyes^14–16^, etc., are continuously increasing.

Dyes are widely utilized in the paper, pulp, paint and textile industries, with a large amount of water demand for washing and cleaning purposes. Based on the Colour Index, currently, more than 10 000 various types of synthetic dyes are available worldwide. In 2014, more than 1.5 million tons of dyes were produced annually. Fifty percent of these dyes are used by the textile industry, where during the colouring process, 1–10% of these dyes are discharged^17,18^. Due to the high solubility and low biodegradability of these compounds, dye-polluted industrial wastewater is one of the most difficult types of wastewater to clean. Remazol Brilliant Violet-5R (RBV-5R), a reactive dye, is used to colour cellulosic fibres. After the colorization process, approximately 10–50% of the initial dye load remains unused^19^.

Biosorption is a promising method for removing persistent compounds, i.e., non-biodegradable compounds. Recently, various materials such as nanocomposites, households and industrial wastes have been used as biosorbents for inorganic and organic contaminant removal^5,11,20,21^. Coconut residual fibres^22^, low-cost activated charcoal from tobacco stems^23^ and eggshell membranes have been successfully used for dye removal^24^.

Due to their porous structure, eggshells have large specific surface areas; therefore, they are excellent biosorbents^25–29^. The eggshell structure is uneven and granulated. One eggshell, which consists of 95–97% calcite (CaCO_3_) crystals, contains approximately 17 000 pores^30,31^. The complex structure of chicken eggshell contains various organic molecules and mineralized components such as calcite (mainly in palisade and mammillary layers), which are combined in several layers. Instead of the simple CaCO_3_ crystal layers, the shell consists of very complex mineral formations in a protein matrix. Five different organic and calcite layers build up chicken eggshell. The eggshell thickness is between 280 and 400 µm^32–35^ and is composed of the inner and outer membrane (70 µm), mammillary layer (100 µm), palisade layer (200 µm), prismatic layer (8 µm) and thin cuticle (10 µm).

Herein, we present the reusability of eco-friendly and inexpensive eggshells for the adsorption of RBV-5R from aqueous effluents, since to the best of our knowledge, this topic is still not investigated. First, the surface structure and morphology of the eggshell was characterized by microscopy (stereo, polarization, SEM), elemental analysis (spot and map EDX measurements), FTIR spectroscopy, Raman spectroscopy and BET-surface density measurements. These ultrastructure studies and elemental analyses give full information regarding the chemical constituents and elemental distribution of the eggshell particles, different layers and the textile dye chemical composition to understand the interaction mechanism between the dye and the biomass. Moreover, optimization of the factors affecting biosorption (dye concentration, biomass weight, particle size, pH, temperature) was performed. Batch adsorption experiments under the optimal conditions were conducted to investigate the adsorption isotherm, kinetic, diffusion and thermodynamic models. An artificial neural network was also employed to build models for maximizing the adsorption capacity of eggshell and to examine optimal processing conditions for the adsorption of RBV-5R onto the eggshell surface.

In addition, the photolytic degradation, photocatalytic decomposition and toxicity of RBV-5R dye were investigated since all these factors strongly influence the environmental impact and toxicity degree of the contaminant at various concentrations.

## Results and discussions

### Microscopy studies

Different microscopic studies were carried out to examine the eggshell structure by comparing the untreated eggshell with that containing adsorbed RBV-5R dye to determine which layers are involved in dye diffusion.

**Figure 1:**
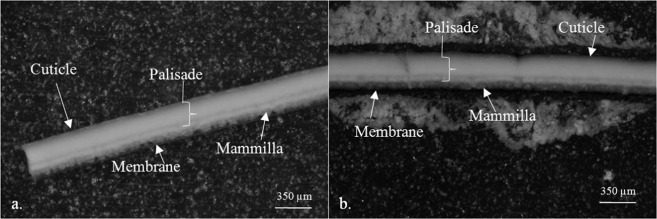
Stereomicroscopic images of thin sections from different structural units (cuticle, palisade, mammilla, membrane layer) of the (**a**) control eggshell and (**b**) eggshell with 2 g/L RBV-5R adsorbed.

**Figure 2:**
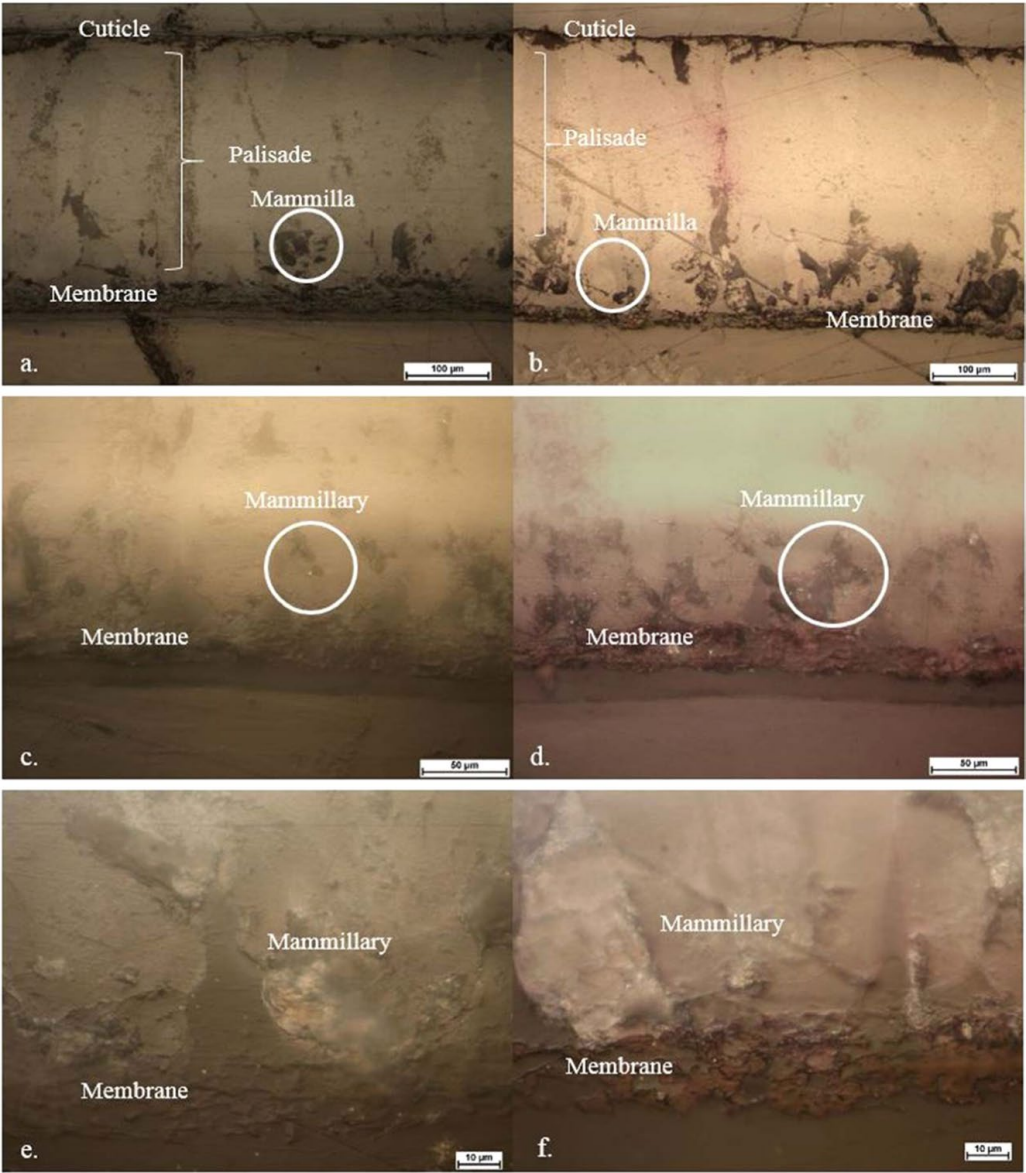
Polarization microscope images of thin sections from (**a**,**c**,**e**) the control eggshell and (**b**,**d**,**f**) eggshell with 2 g/L RBV-5R adsorbed.

Figure 1 shows that the dye-adsorbed samples’ (Fig. 1b) cuticle, membrane and mammilla unit became purple due to adsorption. The palisade layer is only purple if there is a crack on the eggshell surface. The same situation can be seen in the polarization microscope images with reflected light at increasing magnifications from the a-b couple (Fig. 2).

**Figure 3:**
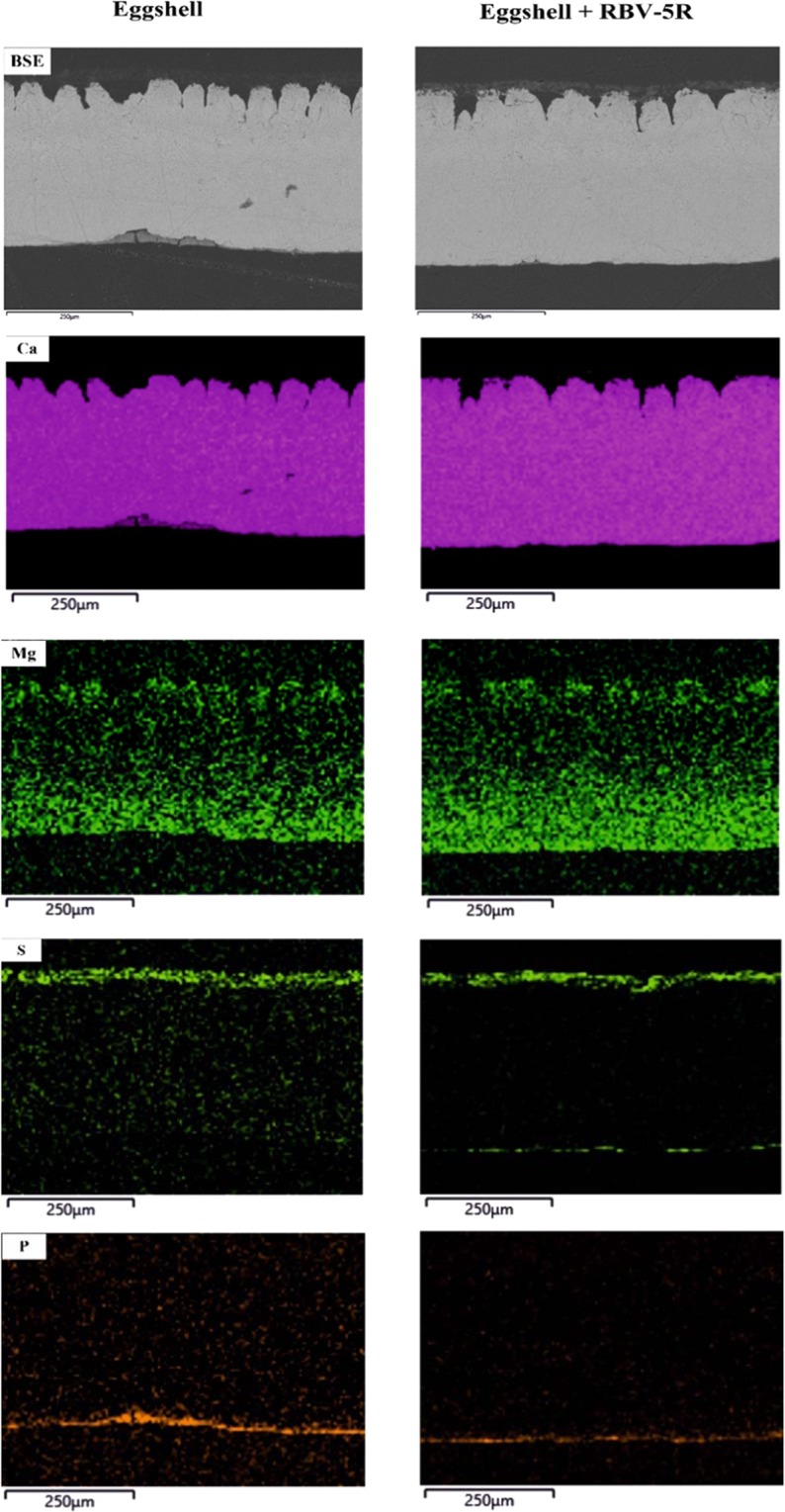
SEM images and EDX elemental maps of an eggshell thin section.

Secondary electron microscopy images, obtained for the control eggshell and the eggshell in the textile-dye solution, display the surface morphology of the eggshell, but the polished surface with the characteristic eggshell layers (membrane, mammillary, palisade and cuticle) was also visualized. Figure 3 presents the elemental distribution of calcium, magnesium, sulphur and phosphorous in the eggshell section. Net intensity maps shed light on the difference in abundances between the minimum and maximum amount of each element. The intensity of the colour is consistent with the concentration of the element in each area.

Mg is highly enriched in the cuticle, and the amount decreases abruptly until the mammillary layer, where it increases, on the other hand relatively homogenous Ca distribution was found in the whole thin sections. Therefore, it can be concluded that eggshell is made of Mg-bearing calcite. The concentration of P is the highest near the outside surface of the eggshell in the cuticle layer. In contrast with that of P, the amount of S is the highest in the inner part of the eggshell in the membrane layer. Since the dye contains sulphur, after adsorption, RBV-5R also appears in the cuticle layer. This mapping method is also used to investigate the absorptivity of different nanomaterials and to determine their composition distribution to better understand the adsorption process^7,36^.

### Elemental composition

Each layer has a different composition. After adsorption, the membrane, mammillary and cuticle layers show the characteristic elements of the dye molecule, namely, N, S and Cu, proving the eggshell’s adsorptive capability. The palisade, composed of columnar calcite crystals, contains Ca, O and C. After the adsorption experiment on the eggshells, the amount of magnesium increased, but the characteristic elements (Cu, S, N, O) of the dye could not be detected, which suggests that the dye did not reach the inner layers of the eggshell, even when a 2 g/L dye solution was used, presumably due to the structure and/or composition of the eggshell layer (Table 1).

**Table 1:** Representative elemental composition of each eggshell layer.

	Membrane wt (%)		Enrichment factor (%)	Mammillary wt (%)		E. factor (%)	Palisade wt (%)		E. factor (%)	Cuticle wt (%)		E. factor (%)
	Control	Dye		C.	D.		C.	D.		C.	D.	
**C**	81.9	61.5	−24.9	18	15.7	−12.9	11.9	12.6	5.7	14.3	25.7	80.2
**N**	0	9.3	>	0	3.1	>	0	0	0	0	4.2	>
**O**	13.5	22.7	67.7	45.3	44.8	−1	47.5	47.6	0.2	47.7	41.7	−12.6
**S**	0	3.2	>	0.1	0.6	358.3	0	0	0	0	1	>
**Ca**	0	1.1	>	35.7	37.1	3.9	40.1	38.7	−3.4	35.6	28.1	−21.2
**Cu**	0	1	>	0	0	0	0	0	0	0	0.4	>

### Raman spectroscopy of each eggshell layer

Eggshell has a complex composition of various organic molecules and mineralized components that are combined through the layers. According to previously published articles, the eggshell membrane contains 75–76% proteins, 4–5% carbohydrates and 20% water. The major protein component has disulphide crosslinks and lysine, while a smaller part is composed of collagen and glycoprotein^37^. The Raman bands of the control and dye-adsorbed thin section eggshell listed in Supplementary Table S1 are characteristic of S–S (507 cm^−1^) bonds, amino acids such as tryptophan (758 cm^−1^), tyrosine (860 cm^−1^), and phenylalanine (1001 cm^−1^), amide groups (1243 cm^−1^), and C–H functional groups (1448 cm^−1^). Raman shifts can also represent carbohydrates such as ribose and xylose at 507, 758, 1115, 1243, 1448 cm^−1^ ^38,39^.

During the daily production of eggs, approximately 6 g of mineral is deposited on a chicken eggshell^40^. The palisade layer is mostly composed of CaCO_3_, and its Raman bands are located at 282, 712 and 1087 cm^−1^.

The spongy and organic cuticle covers the entire outer layer of the eggshell (Fig. 1). Since the outer layer of the eggshell, which is attached to the lime shell, forms the top layer, gas is exchanged by osmosis, and there are no pore channels. The cuticle is composed of organic matter: 80–95% protein (water insoluble), 4–5% carbohydrates, 2.5–3.5% lipids and 3–3.5% ash^33,35,37,41^. Raman shifts at 1115 and 1274 cm^−1^ in our samples certify the presence of neutral fats^42^ such as triacylglycerol, and bands at 860 and 1274 cm^−1^ indicate the presence of amide bonds (specific to albumen and collagen) and tyrosine amino acids. The presence of carbohydrates is proven by bands at 503, 606, 1115 (xylose), 633, 1115, and 1274 cm^−1^ (ribose) as well as 356 and 633 (lactose).

After dye adsorption, new bands appear in each layer at 520, 580/581, 996, 1258/1261, 1304, 1332, 1403 and 1437 cm^−1^ that are specific to the C–H, C–S and N = N functional groups of the dye.

### Characterization of eggshell powder

Total surface area (St), pore volume (Vp) and pore radius (Rm) were calculated, and eggshell density was also determined with ethanol. The surface area of 160 µm pore size eggshell powder was 1.8 m^2^/g, the pore volume was 0.01 cm^3^/g and the density (ρ) was 1.3 g/cm^3^. The pore radius presented a multimodal mesoporous-macroporous pore size distribution, with three different size regions: 20–30 Å, 60–80 Å and 200 Å. For eggshell powder with a 315 µm pore size, the surface area was below 1 m^2^/g and could not be evaluated.

### Photolytic degradation and photocatalytic decomposition

During light exposure, the dye concentration remained constant, suggesting that RBV-5R dye shows no photolytic degradation. It can be concluded that during the adsorption process, RBV-5R was adsorbed on the surface of the eggshell. RBV-5R decomposition was analysed by a P25 (TiO_2_) catalyst, resulting in an efficiency of 99.82% after 120 minutes of exposure, while the dye was oxidized to CO_2_ and H_2_O (Supplementary Fig. S1). Similar results were obtained by Bizani *et al*.^43^, who investigated the oxidation of two reactive azo dyes (Cibacron Red FNR, Cibacron Yellow FN2R) using two types of TiO_2_ catalysts (P25, Hombicat UV-100). Under these experimental conditions, the dyes decomposed by almost 100% after 100 minutes of illumination with the P25 catalyst, whereas when using Hombicat UV-100, less time (50 minutes) was necessary. Hasnat *et al*.^44^ investigated TiO_2_ and ZnO catalysts’ photodegradation ability on erythrosine synthetic anionic dye; TiO_2_ proved to be the better catalyst.

### Effect of the initial parameter change

A high variety of household and agricultural wastes have been used as biosorbents for the removal of synthetic RBV-5R dye from aqueous solutions under different experimental conditions. Hashemian *et al*.^45^ used activated charcoal of orange peel origin, whereas Bello and Ahmad^46^ used periwinkle shells as an activated charcoal source, where the RBV-5R dye removal yield was in the range of 30 to 92%.

**Figure 4:**
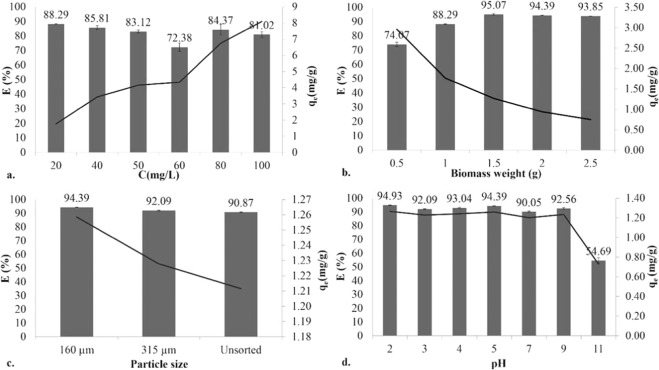
Effect of (**a**) initial dye concentration (C_i_ = 20–100 mg/L, 1.5 g biomass, 160 µm, 700 rpm, pH = 6.0 ± 0.2, T = 20 ± 1 °C), (**b**) biomass weight (C_i_ = 20 mg/L, 160 µm, 700 rpm, pH = 6.0 ± 0.2, T = 20 ± 1 °C), (**c**) particle size (C_i_ = 20 mg/L, 1.5 g biomass, 700 rpm, pH = 6.0 ± 0.2, T = 20 ± 1 °C), and (**d**) pH (C_i_ = 20 mg/L, 1.5 g biomass, 160 µm, 700 rpm, T = 20 ± 1 °C), where standard deviations were calculated from measurements from three parallel experiments.

The effect of the initial dye concentration on the uptake of RBV-5R dye by our eggshell waste was studied using dye concentrations ranging between 20 and 100 mg/L. With increasing initial concentration, the adsorption capacity also increased, whereas the efficiency decreased (Fig. 4a).

The effect of the amount of eggshell on both the biosorption capacity and the percentage of RBV-5R dye removal was studied at five different adsorbent dosages (0.5–2.5 g). The figure shows that by increasing the adsorption dosage, the removal efficiency also increased until 1.5 g/100 mL, but after that, saturation was reached (Fig. 4b).

We tested the effect of particle size on the biosorption process. The rate of paint removal shows an increasing trend as the particle size decreases. Both the biosorption capacity and the efficiency increase with increasing particle size. The highest efficiency (94%) was obtained with eggshells with a particle size of 160 µm (Fig. 4c).

Dye biosorption is a pH-dependent process. Therefore, the initial pH of the solution can significantly influence not only the chemical structure of the dyes but also the eggshell surface charge. The effect of the pH was measured in a pH range from 2 to 11. Reactive dyes are anionic in nature, so they are expected to be adsorbed much better in acidic media (E > 94%) (Fig. 4d).

The influence of temperature on the adsorption efficiency and capacity of RBV-5R was studied, whereby thermodynamic parameters were calculated. Our results show that the adsorption capacity and the percentage of RBV-5R biosorption on eggshell biomass is an exothermic process because the efficiency decreased with increasing temperature. The maximum efficiency was 94.4% at 20 °C, while 92.4% at 30 °C and 89.7% at 40 °C. A similar trend can be observed in the case of the adsorption capacity.

Thermodynamic parameters were calculated for C_i_ = 20 mg/L dye solution, 1 g biomass, 160 μm eggshell powder size, 700 rpm agitation speed, and pH = 6.0 ± 0.2. From the results, we can see that the Gibbs free energy (ΔG) decreases with increasing temperature (T: 293 K = 0.375 kJ/mol, T: 303 K = −0.480 kJ/mol, T: 313 K = −1.336 kJ/mol), indicating that adsorption is a spontaneous process. The calculated value of the enthalpy (ΔH = 25.44 kJ/mol) is less than 85 kJ/mol, which proves that adsorption involves a physical process. The positive entropy value (ΔS = 0.086 J/mol∙K) suggests increased randomness at the solid/solution interface^46,47^.

It can be concluded that the adsorption had the highest efficiency of 95% when the initial concentration was 20 mg/L and 1.5 g/100 mL biomass was added to the solution, which was powdered to 160 μm. The adsorption was carried out under constant shaking at 700 rpm, without pH adjustment and at room temperature (pH = 6.0 ± 0.2, T = 20 ± 2 °C).

### Adsorption equilibrium isotherm models

The adsorption isotherm models describe the performance and interaction of the adsorbate with the adsorbent. To define the equilibrium nature of adsorption, four isotherm models (Langmuir, Freundlich, Temkin, Dubinin-Radushkevich) were used. Based on the correlation coefficients (Langmuir: 0.945, Freundlich: 0.839, D-R.: 0.563 and Temkin: 0.709, shown in Supplementary Table S2) obtained from plotting the linearized isotherms, the Langmuir isotherm model fits best, with high accuracy. These isotherms are characterized by specific constants that not only express the surface properties of eggshells but also describe the adsorption type and its nature. Because the Temkin constant *B* (B = 4 × 10^−5^ J/mol) is less than 20 kJ/mol and the energy *E* (E = 0.5 kJ/mol) is less than 8 kJ/mol, we can confirm that adsorption occurs by physisorption, forming weak van der Waals interactions in a single-layer adsorption surface with equivalent binding sites. The Langmuir isotherm model indicates that adsorption took place in the specific homogenous sites of the eggshell; it assumes that once an adsorbed molecule occupies the available eggshell adsorption sites, no more adsorption can occur on the surface sites of the material. Furthermore, we reach maximum adsorption when a saturated monolayer appears on the eggshell surface. The model cannot provide a solid and clear depiction or understanding of the mechanical phenomena, but it gives the adsorption capacity value, which is 9.94 mg/g in this study^4^.

### Adsorption kinetics and diffusion models

To better understand the mechanism of RBV-5R adsorption on eggshell and to find the best-fitting model to describe our obtained data, various kinetic models were applied. The kinetic adsorption models establish the effect of time on adsorption and determine the rate of RBV-5R dye removal. Experimental data were analysed using pseudo-first-order (Lagergen) and pseudo-second-order (Ho & McKay) kinetic models. The calculated obtained linear regression coefficient values for the pseudo-second-order kinetic model were greater than those for the pseudo-first-order kinetic model (Supplementary Table S3); therefore, the former model more appropriately describes the mechanism of RBV-5R adsorption on eggshell.

Intra-particle and liquid film diffusion rate constants and linear regression coefficients were calculated. Based on the literature data, three regions can be observed if we study intra-particle diffusion: (1) diffusion of RBV-5R molecules through the solution to the external surface of eggshell; (2) transport of RBV-5R molecules to the intra-particle active sites; and (3) adsorption of RBV-5R molecules to the eggshell intra-particle active sites. The obtained results (*D*-pore diffusion coefficient values ranging between 1.7×10^–10^ and 3.6×10^–10^ cm^2^/s), as well as the fact that the intercept points do not pass through the origin, lead to the conclusion that the two diffusion models are not rate-determining steps, and only the biosorption process affects adsorption speed (Supplementary Table S4).

### Scanning electron microscopic and energy-dispersive X-ray spectroscopic characterization of the biomass

**Figure 5:**
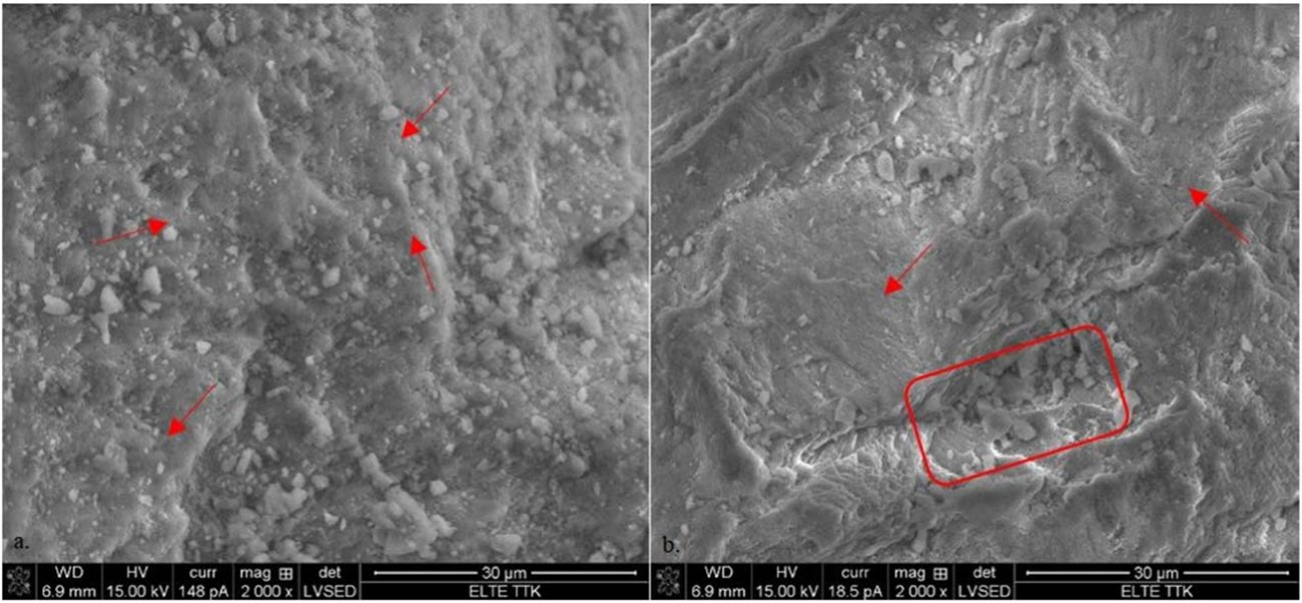
SEM micrographs of powdered eggshell (**a**) control and (**b**) eggshell with 100 mg/L RBV-5R adsorbed.

The surface characterization (morphology and texture) of the homogenous eggshell powder before and after adsorption was determined by scanning electron microscopy. Images were taken at 2 000× magnification, where one can observe the macroporous structure and the rough and dense texture of the control sample of powdered eggshell (Fig. 5a). Moreover, the high porosity of the eggshell powder particles is also shown, which can be identified as gas exchange pores. This structure ensures the adsorption capacity of the eggshell, as it can accumulate dye in the pores. After contacting the dye (Fig. 5b), the surface became smoother as if it had been polished, with apparently fewer and smaller pores.

EDX measurements on powdered eggshell were performed before and after adsorption of the dye. The results obtained are the means and standard deviations of 17 analyses (C_RBV-5R_ = 100 mg/L, 160 μm). The control sample contains a high proportion of carbon (23 ± 9 wt%), oxygen (44 ± 1 wt%) and calcium (29 ± 10 wt%, Supplementary Table S5). The RBV-5R dye consists of carbon (33.5 ± 2 wt%), oxygen (38 ± 11 wt%), nitrogen (2.4 ± 5 wt%), sulphur (0.6 ± 0.8 wt%) and in traces copper (0.08 ± 0.15 wt%), which appear in the eggshell composition after biosorption. The appearance of S and Cu and the increased amount of N clearly demonstrate the ability of the eggshell to adsorb RBV-5R.

### Fourier transformation infrared spectroscopy

**Figure 6:**
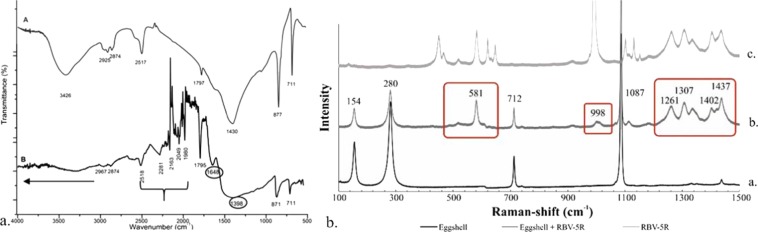
(**a**) FTIR spectra of the (**a**) control eggshell and (**b**) eggshell with 2 g/L RBV-5R adsorbed, (**b**) Raman spectra of the (**a**) control eggshell, (**b**) eggshell with 2 g/L RBV-5R adsorbed, and (**c**) dye.

Functional groups present on eggshell powder before and after RBV-5R dye adsorption were also identified by FTIR spectroscopy. Figure 6a shows characteristic FTIR spectra, providing information on the binding nature of eggshell with the dye. Following adsorption, new bands appear, including at 1398 cm^–1^, representing sulfonic groups characteristic of the dye; at 1648 cm^–1^, indicating naphthalene; and between 1980 and 2281 cm^–1^, representing the dye fingerprint zone. Stretching vibrations appear above 2967 cm^–1^, specific to phenolic O–H, secondary amine N–H, and aromatic C–H bonds^48–50^.

### Raman spectroscopy

To determine the material structure of eggshell powder before and after dye adsorption, vibrational Raman spectroscopic measurements were carried out. Information on the eggshell adsorbent and the spectra obtained in the Raman shift range of 100–1500 cm^−1^ are shown in Fig. 6b; In the spectrum of the untreated and treated eggshell, the typical bands of calcite (CaCO_3_) mentioned in the literature^51,52^ are also found at 154–155, 280–281, 712, and 1087 cm^−1^. The spectrum of dye-adsorbed eggshell contains some characteristic bands of the dye as well (with a smaller or greater intensity, namely, 520, 581–582, 992–998, 1261, 1307, 1334, 1402 and 1437 cm^−1^). The newly emerging bands typically correspond to aliphatic C–S bonds and N = N bonds^53^.

### Possible adsorption mechanism

Based on our current knowledge, no binding process or mechanism between the eggshell and the dye has been described explicitly in any research article. Relying on our results and the little information found in the literature, we formulate our ideas about possible processes. The mechanism of RBV-5R dye, as with any dyestuff’s adsorption on eggshell or different adsorbent surfaces, highly depends on the physical and chemical characteristics of the adsorbent and dye. Therefore, a high range of parameter changes and analytical measurements were carried out, as discussed in the previous sections.

The initial pH of the solution is an important factor for adsorption processes. The pH can alter the chemical balance of the ions present both in the RBV-5R dye and eggshell adsorbent; thus, the pH influences the electrostatic interactions between the dye and sorbent. The organic matter of eggshell comprises various functional groups, such as hydroxyl, amine, and carbonyl groups. When the eggshell powder was mixed with the dye solution, calcium salts may partially dissolve and release Ca^2+^, HCO_3_^−^, CO_3_^2−^ and OH^−^ ions, which can form a negatively surfaced charge^54,55^. In acidic media (HCl), the eggshell surface exhibits a positive charge, so there is a high electrostatic attraction between the eggshell powder and the anionic, negatively charged dye (this dye contains aromatic rings such as phenolic OH and sulfonate SO_3_^−^ groups that ionize in aqueous solution and form coloured anions^49^). By adding NaOH, the number of negatively charged sites of eggshell increases, resulting in electrostatic repulsion.

The adsorption mechanism was studied by isotherm, kinetic and diffusion models. Since in our experimental conditions the Langmuir isotherm showed a better fit, adsorption takes place on a homogenous surface, and monolayer adsorption occurs. In this case, we can calculate the R_L_ separation parameter^55^; the R_L_ value indicates the type and favourability of the isotherm to be irreversible (R_L_ = 0), favourable (0 < R_L_ < 1), linear (R_L_ = 0) or unfavourable (R_L_ > 1). We found that 0.1 < R < 0.35, which suggested favourable adsorption; however, R_L_ values close to the lower acceptable range suggest high irreversibility. Constant calculations from the Temkin and Dubinin-Radushkevich models, as mentioned before, suggest the appearance of physisorption where weak van der Waals interactions are formed in a single-layer adsorption surface with equivalent binding sites. The physical nature and spontaneous and random characteristics of adsorption are suggested by the calculated thermodynamic parameters.

FTIR and Raman analyses (both for powdered eggshell and for different layers of eggshell) proved the presence of specific functional groups from organic and inorganic compounds such as ^−^OH, C=O, =CH_2_, aromatic, –SH and –COOH groups. All these surface functional groups indicate that eggshell exhibits a moisture adsorption capability, which makes this material a potential adsorbent by participating in adsorption. FTIR spectra of RBV-5R showed bands that correspond to phenolic O–H, aromatic C–H, amide N–H, aliphatic C–H_n_, amide C = O, C = C, N = N, R–SO_3_^−^ and C–S functional groups^49^.

The results obtained from the pH studies and FTIR/Raman analyses indicate that adsorption occurred mostly via electrostatic attraction, where the protonated eggshell surface attracted the anionic dye sulfonate group. Considering two previous articles^49,56^, there is a possibility of three interactions between the dye and adsorbent: hydrogen bonding between hydroxyl groups of eggshell and electronegative residues of RBV-5R; ionic interactions at pH values where surface charge is neutral and physisorption occurs; and π-electron resonance.

### Eggshell as an adsorbent – advantages and disadvantages

Since a limited number of studies have been reported on the adsorption of Remazol dyes with untreated eggshell, this economically feasible adsorbent is feasible for Remazol dye removal from contaminated water.

Eggshell as waste contributes to pollution^57^ when disposed of in landfills due to its rich protein content because it serves a source for biocontamination. The messy holding containers in which eggshell waste is stored can have an unpleasant odour, which can affect nearby neighbourhoods, causing air pollution.

The eggshell adsorbent employed was used without any physical or chemical treatment, avoiding special sophisticated methods, instruments, or controlled conditions (pH, temperature). The material was efficient even for high dye concentration elimination (E = 81% in case of C_i_ = 100 mg/L), where the adsorption capacity was 8.1 mg/g. Particle size was the most significant influencing parameter regarding the efficacy of the adsorbent.

Compared with other adsorbents such as nanomaterials, eggshell can be slower and harder to recover in its original form^10,58–60^. A comparison is almost impossible due to the different base materials of the adsorbents as well as different contaminants and processing conditions. On the other hand, we previously investigated the adsorption of this same dye (RBV-5R) with calcined eggshell under the same conditions (C_i_ = 20 mg/L, 1.5 g/100 mL biomass, 160 μm, 700 rpm, pH = 6.0 ± 0.2, T = 20 ± 2 °C), which exhibited an adsorption efficiency of 96.83% and adsorption capacity of 1.29 mg/g, reaching equilibrium within 30 minutes^27^.

Overall, in addition to the disadvantages, the use of eggshells as an adsorbent will not only reduce the pollution effect of the waste but also help in the water cleaning process as a new value-added product. The practice of using untreated eggshell waste as a low-cost adsorbent for wastewater treatment could contribute to a more sustainable and successful management of this biowaste.

### Ecotoxicological test-seedling growth test

Root growth inhibition ecotoxicological tests were carried out to study the effect of the dye on lettuce (*Lactuca sativa*) and mustard (*Sinapis alba*) seedling growth. The data listed in Supplementary Table S6 show the results of two parallel experiments as required by the standard used. It is clear that root growth inhibition is plant dependent. Even at low dye concentrations, more than 50% inhibition occurred in lettuce germination. A similar method was used in our previous work in which we studied the effect of two anionic and two cationic dyes on lettuce and mustard seeds^28^. The results showed that the shooting of germinated seeds (clover, wheat, lettuce, tomato) is dye dependent^61^. After a 2-day examination, the irrigated seed germination decreased with increasing concentration. On the other hand, no toxicity to Acid Blue 40 and Acid Red 151 after 15 minutes of electrolytic treatment was found^61^.

### Artificial neural network

Since OUTPUT = TARGET + 0.0100 (received from the program and shown in Supplementary Fig. S2a), our network has a good performance, and it predicts with high precision. The network topologies of the input, middle and output components are shown in Supplementary Fig. S2b. The network exhibited a significant weight distribution of negative values for the initial concentration and the pH and eventually the time; simultaneously, high positive input weights were observed for the initial concentrations, biomass weight and pH. Our results show a succession of sensitivity, which determines the network’s main weights, giving a hierarchy of the most influential parameters: initial concentration → pH → biomass weight → contact time → particle size → temperature. Similar results were reached by^48,62,63^.

## Conclusion

The structure of eggshell was studied by using different microscopic techniques. The elemental composition (distribution maps and SEM-EDX measurements) proved the eggshell adsorptive capability because characteristic elements of the dye molecule, namely, N, S and Cu, appeared in the membrane, mammillary and cuticle layers. Raman spectroscopy of the eggshell layers certifies that eggshell has a complex composition of various organic molecules such as proteins, lipids and carbohydrates and mineralized components (Mg-calcite) that are combined throughout the four layers.

The present study proves that eggshell household waste can be used with high efficiency as an adsorbent for the removal of RBV-5R dye from aqueous solutions. Investigating the dye’s photocatalytic decomposition by a TiO_2_ (P25) catalyst, >99% efficiency was reached within 120 minutes, whereby the dye was oxidized to CO_2_ and H_2_O.

In our study, the optimal parameters for dye removal were C_i_ = 20 mg/L, 1.5 g biomass, grain size 160 μm, 700 rpm, pH = 6.0 ± 0.2, and T = 20 ± 2 °C, where E% = 95. The isotherm models fit the Langmuir isotherm model well, indicating that monolayer adsorption occurs. Under our experimental conditions, the adsorption process can be described by the pseudo-second-order kinetic model. Neither intra-particle diffusion nor film diffusion affects the rate of adsorption.

The SEM, EDX, FTIR and Raman analyses proved that RBV-5R was present in the eggshell after the adsorption process because aggregates appeared on the surface of the eggshell, the amounts of characteristic elements such as S, N and Cu increased, and new bands and shifts in the spectra of the treated samples appeared, corresponding to phenolic O–H, secondary amine N–H, aromatic C–H, N = N and C–S bonds.

With the help of the initial pH change, isotherm, kinetic, and thermodynamic models along with analytical methods, we proposed three possible explanations of the adsorption mechanism and interactions between the dye and eggshell.

According to the ANN model results, under our experimental conditions, the initial dye concentration is the most important influential parameter.

## Methods

### Reactive dye

The reactive azo dye Remazol Brilliant Violet-5R (RBV-5R, chemical formula: C_20_H_16_N_3_Na_3_O_15_S_4_, MW = 734.58 g/mol) was obtained from DyStar Singapore Pte. Ltd., Singapore and was used without further purification. The product contains approximately 3.7% copper. A 2 g/L stock solution was prepared with deionized (MilliQ) water by dissolving the dark blue to dark violet powder, and then, it was diluted to the desired concentration with deionized water in a 100-mL volumetric flask. The dye has been used in the textile industry for cotton, silk and linen dyeing and printing. As a reactive dye, it is used to dye cellulosic fibres, which contain N = N double bonds.

### Preparation of adsorbent

Eggshells were collected from kitchen waste. To prevent decomposition and to remove dirt particles, the samples were washed several times with tap water, subsequently with deionized water, and finally dried in a drying cabinet at 80 °C to reach a constant weight. The dried eggshells were used in two forms for different reasons: (1) approximately 0.5 cm diameter eggshell units for the structure study and (2) powdered eggshells for adsorption study.

The dried eggshells were powdered in a mortar using an electric grinder. Finally, the different particle sizes were separated with a sieve set. Until usage, the eggshells were stored in airtight boxes. Eggshell powder ground to different particle sizes (micrometre scale) was used as an adsorbent without any physical or chemical pre-treatment.

To study the eggshell structure before and after dye adsorption, thin sections were made perpendicular to the eggshell structure using two-component epoxy resin (Buehler EpoThinTM2). Following the preparation of the sample, the structure of the eggshell was microscopically studied.

### Photolytic degradation and photocatalytic decomposition

To investigate the photochemical behaviour of the dye, photolytic and photocatalytic (TiO_2_) degradation was studied. The photocatalytic activity was determined by using a 6×6 W fluorescent ultraviolet light source with a low-pressure mercury lamp at λ_max_ = 365 nm, thermostated at 25 °C.

### Batch experiments

The sorption of RBV-5R dye on the untreated eggshell surface was investigated in aqueous solutions in a 250 mL Erlenmeyer flask containing 100 mL dye solution and various amounts of eggshell powder. Experiments were conducted until the concentration reached equilibrium. The concentration of RBV-5R was continuously measured by an Agilent Cary 60 UV-Vis spectrophotometer at λ_max_ = 553 nm. A calibration curve quantitative measuring technique was used (R^2^/n = 0.998/8).

### Optimization of initial parameters

To examine the effect of the initial RBV-5R concentration and contact time on eggshell adsorption, 100 mL volumes of solutions at different initial concentrations (20–100 mg/L) of the dye were mixed with 1 g biomass in a 250 mL Erlenmeyer flask. The constant parameters are as follows: particle size of 160 µm, room temperature of 20 °C, 700 rpm agitation speed, and initial pH of 6.

The effect of biomass weight was studied by mixing 100 mL solutions containing 20 mg/L RBV-5R dye with 0.5/1/1.5 g biomass from eggshells (160 µm particle size) at room temperature, with an agitation speed of 700 rpm, without pH adjustment.

To study the effect of the particle size of eggshells, 100 mL RBV-5R dye solution (20 mg/L concentration) was prepared. Then, 1.5 g eggshell with different particle sizes (<=160 µm, 160–315 µm, unsorted particle size range) was added to the solution and agitated at constant agitation speed (700 rpm) without temperature or pH adjustment.

The effect of the pH on the adsorption of RBV-5R was studied by mixing 1.5 g eggshells of 160 µm particle size with 100 mL 20 mg/L RBV-5R solution in a pH range between 2 and 11 at room temperature. The pH was adjusted with 1 N HCl or NaOH solutions.

The effect of temperature on the adsorption process was examined by using an IKA C-MAG HS7 digital multi-magnetic shaker by varying the solution temperature (20, 30, 40 °C), mixing 100 mL 20 mg/L RBV-5R with 1.5 g 160 µm particle size eggshells, without pH adjustment.

After each condition measurement, the received data were further analysed, and the adsorption efficiency and the amount of RBV-5R adsorbed on the eggshell surface were calculated based on previously described methods, moreover isotherm, kinetic and diffusion models were calculated^4,12,29,64^.

### Analytical methods

Powdered eggshell was further studied with Oxford Instruments EDS Analysis System Inca 300 (UK) to examine its elemental composition^27–29^. Fourier transform infrared spectroscopy data were obtained using a JASCO 615 FTIR spectrophotometer in the wavelength range of 500–4000 cm^–1^, and the observed bands were analysed by using ORIGIN PRO 8.5 software. These studies were carried out at National Institute for Research and Development of Isotopic and Molecular Technologies, INCDTIM Cluj-Napoca, Romania.

The structure of the thin sectioned eggshell was studied using stereomicroscope (Nikon SMZ1000 and Nikon D5000), polarization microscope (Nikon Eclipse LV100 POL), scanning electron microscope – SEM (FEI Quanta 3D), and EDX (Hitachi TM4000 Plus SEM equipped with Quantax 75 SDD EDS system) measurements were also performed on each layer. SEM images were performed using 15 kV high voltage and 18–150 pA beam current under low vacuum chamber pressure (80–100 Pa water steam environment). Spot EDX measurements were carried out using 20 kV high voltage and 4 nA beam current. In order to visualize the distribution of different elements within eggshell profiles (Ca, Mg, S, P) EDX elemental maps were carried out using 15 kV high voltage and 2.4 nA beam current. Analyzes were taken in the Research and Instrument Core Facility of the Faculty of Science, Eötvös Loránd University, Budapest.

Total surface area (St), pore volume (Vp) and pore radius (Rm) were obtained from N_2_ adsorption–desorption isotherms (measured at −196 °C) using the BET model for St determination and the Dollimore–Heal method for Vp and Rm. The isotherms were recorded using a Sorptomatic 1990 apparatus (Thermo Electron Corporation). Prior to determination, the samples were degassed at 150 °C under vacuum (approximately 1 Pa) for 3 hours to remove the physically adsorbed impurities from the surface. No pressure variation was observed during 1 hour at the end of sample degassing^27^. The eggshell density was determined with ethanol using a pycnometer^65^.

Raman microspectroscopic measurements were carried out at the Research and Instrument Core Facility of the Faculty of Science, Eötvös Loránd University, Budapest. To study the eggshell powder (dye treated and control) and dye powder, we used a confocal HORIBA Labram HR (high-resolution) spectrometer with Nd:YAG laser (λ = 532 nm) excitation and 1800 grooves/mm optical grating. The laser power was 130 mW, and the laser spot diameter was ~1.5 μm^66^.

### Ecotoxicological test-seedling growth test

Using the MSZ21978/8–85 standard, the effect of the dye on the growth of lettuce and mustard seeds was investigated^28,29^. Based on the given standard, the experiments were carried out two times in autoclaved Petri dishes, where 5–5 mL dye solution at different concentrations was measured with 25 seeds. The samples were kept in the dark for 72 hours at T = 20 ± 2 °C. The root growth inhibition was calculated using the following equation with the measured lengths of the seeds, where the length was measured by a digital calliper:

1 $${\rm{X}}=\frac{{\rm{K}}-{\rm{M}}}{{\rm{K}}}\times 100$$

where *X* is the root growth inhibition (%), *K* is the root length (mm) of the control seeds, and *M* is the contaminated seed root length (mm).

### Artificial neural network (ANN)

An artificial neural network (ANN) is a statistical modelling method (functioning as a black box) that helps to understand the behaviour of a system by knowing input data and predicting output results. Six input parameters (i.e., initial concentration, biomass weight, particle size, pH, temperature, contact time) were given to the EasyNN software program package, thereby resulting in a 6:7:1 network structure^48,62,63^.

## Supplementary information

Supplementary document.

## Data Availability

All data generated or analysed during this study are included in this published article (and its Supplementary Information files).
