# Supplementary material for: Adsorption of Remazol Brilliant Violet-5R Textile Dye from Aqueous Solutions by Using Eggshell Waste Biosorbent
Source: Sci Rep. 2020 May 20;10:8385. doi: 10.1038/s41598-020-65334-0 (PMC7239865; doi:10.1038/s41598-020-65334-0)
Supplement: Supplementary file 1 — Supplementary document. [file 41598_2020_65334_MOESM1_ESM.docx]

**Supplementary document - tables and figures**

**Adsorption of Remazol Brilliant Violet-5R Textile Dye from Aqueous Solutions by Using Eggshell Waste Biosorbent**

Eszter Rápó^1,2^, László Előd Aradi^3^, Ábel Szabó^3^, Katalin Posta^2^, Robert Szép^4^, Szende Tonk^1*^

^1^Environmental Science Department, Sapientia Hungarian University of Transylvania, Calea Turzii no. 4, 400193 Cluj-Napoca, RO

^2^Institute of Genetics, Microbiology and Biotechnology, Szent István University Páter Károly no. 1, 2100 Gödöllő, HU

^3^Lithosphere Fluid Research Lab, Eötvös Loránd University, Pázmány Péter sétány no. 1/c, H-1117 Budapest, HU

^4^Faculty of Technical and Social Sciences, Sapientia Hungarian University of Transylvania, Piaţa Libertăţii no. 1, 530104, Miercurea-Ciuc, RO

**CORRESPONDING AUTHOR**

tonk.szende@sapientia.ro

**Table S1.** Raman shifts of control and dye adsorbed thin section eggshells

| **Eggshell layers** | **BANDS (cm^-1^)** | | **Membrane** | 355 | ­- |
| --- | --- | --- | --- | --- | --- |
|  | **Control** | **Dye adsorbed** |  | 507 | ­- |
| **Palisade** | 282 | 281 |  | ­- | 520 |
|  | 712 | 714 |  | ­- | 581 |
|  | 1087 | 1086 |  | ­- | 622 |
|  | ­- | 1332 |  | 758 | ­- |
|  | ­- | 1437 |  | 860 | - |
| **Cuticle** | 356 | 355 |  | ­- | 912 |
|  | 503 | 501 |  | ­- | 996 |
|  | ­- | 580 |  | 1001 | ­- |
|  | 606 | ­- |  | 1011 | ­- |
|  | 633 | ­- |  | 1086 | ­- |
|  | 666 | ­- |  | 1115 | 1111 |
|  | 813 | 811 |  | 1160 | ­- |
|  | 860 | 859 |  | 1243 | ­- |
|  | 1115 | 1114 |  | ­- | 1258 |
|  | 1274 | ­- |  | ­- | 1302 |
|  | -­ | 1261 |  | ­- | 1332 |
|  | ­- | 1305 |  | ­- | 1401 |
|  | ­- | 1335 |  | ­- | 1433 |
|  | ­- | 1403 |  | 1448 | ­- |

**Fig. S1**. Photolytic degradation, photocatalytic decomposition of RBV-5R (C_i_=50 mg/L, m_P25_=0.5 g/L, T=25 ^o^C)

**Table S2**. Adsorption isotherm models calculated constants for RBV-5R adsorption onto eggshell surface (C_i_=20–100 mg/L, 1 g biomass, 160 μm, 700 rpm, pH=6.0±0.2, T=20±2 ^o^C)

| **Langmuir** | | | **Freundlich** | | | **Dubinin-Radushkevich** | | | **Temkin** | | |
| --- | --- | --- | --- | --- | --- | --- | --- | --- | --- | --- | --- |
| **K_L_** | **q_max_** | **R^2^** | **n** | **K_f_** | **R^2^** | **Β** | **E** | **R^2^** | **A_T_** | **B** | **R^2^** |
| **(l/mg)** | **(mg/g)** |  |  | **(mg^(1−1/n)^l^1/n^/g)** |  | **(mol^2^ kJ^2^)** | **(kJ/mol)** |  | **(l/g)** | **(J/mol)** |  |
| 0.093 | 9.935 | 0.945 | 1.589 | 1.09 | 0.839 | 2×10^-6^ | 0.5 | 0.563 | 2.4 | 4×10^-5^ | 0.709 |

**Table S3**. Adsorption kinetics (C_i_=20–100 mg/L, 1 g biomass, 160 μm, 700 rpm, pH=6.0±0.2, T=20±2 ^o^C)

| **RBV-5R** | | **Pseudo-first-order** | | | **Pseudo-second-order** | | |
| --- | --- | --- | --- | --- | --- | --- | --- |
| **C** | **q_e_ (exp)** | **k_1_** | **q_e_ (calc)** | **R^2^** | **k_2_** | **q_e_ (calc)** | **R^2^** |
| **(mg/L)** | **(mg/g)** | **(1/min)** | **(mg/g)** |  | **(g/mg×min)** | **(mg/g)** |  |
| 20 | 2.34 | 1.04×10^-2^ | 1.546 | 0.970 | 1.30×10^-2^ | 1.988 | 0.980 |
| 40 | 5.68 | 1.31×10^-2^ | 3.136 | 0.987 | 1.46×10^-2^ | 3.634 | 0.995 |
| 50 | 8.44 | 1.45×10^-2^ | 4.918 | 0.884 | 0.74×10^-2^ | 4.371 | 0.993 |
| 60 | 16.57 | 0.97×10^-2^ | 4.379 | 0.942 | 0.43×10^-2^ | 4.715 | 0.981 |
| 80 | 12.50 | 0.94×10^-2^ | 4.802 | 0.960 | 0.62×10^-2^ | 6.978 | 0.996 |
| 100 | 18.98 | 1.14×10^-2^ | 8.710 | 0.896 | 0.29×10^-3^ | 8.666 | 0.987 |

**Table S4**. Diffusion models (C_i_=20–100 mg/L, 1 g biomass, 160 μm, 700 rpm, pH=6.0±0.2, T=20±2 ^o^C)

| **RBV-5R** | | **Intra-particle diffusion** | | | **Liquid film diffusion** | | |
| --- | --- | --- | --- | --- | --- | --- | --- |
| **C (mg/l)** | **D (cm^2^/s)** | **k_ip_ (mg/g∙min^1/2^)** | **intercept** | **R^2^_ip_** | **k_fd_ (1/min)** | **intercept** | **R^2^_fd_** |
| 20 | 1.77×10^-10^ | 0.101 | 0.212 | 0.917 | 0.007 | -0.29 | 0.904 |
| 40 | 3.63×10^-10^ | 0.152 | 0.928 | 0.915 | 0.014 | -0.31 | 0.988 |
| 50 | 2.21×10^-10^ | 0.193 | 0.684 | 0.939 | 0.012 | -0.07 | 0.890 |
| 60 | 1.38×10^-10^ | 0.196 | 0.511 | 0.980 | 0.003 | -0.25 | 0.959 |
| 80 | 2.94×10^-10^ | 0.257 | 1.562 | 0.937 | 0.005 | -0.45 | 0.949 |
| 100 | 1.70×10^-10^ | 0.352 | 1.308 | 0.963 | 0.004 | -0.33 | 0.959 |

**Table S5**. Elemental composition of eggshells, results obtained were computed from 17 analyses (C_RBV-5R_=100 mg/L, 160 μm)

| **Elements** | **wt. (%) eggshell** | **wt. (%) eggshell + RBV-5R** |
| --- | --- | --- |
| **C** | 23 ± 9 | 33.5 ± 2 |
| **N** | 0.3 ± 0.7 | 2.4 ± 5 |
| **O** | 44 ± 1 | 38 ± 11 |
| **S** | 0.2 ± 0.3 | 0.6 ± 0.8 |
| **Ca** | 29 ± 10 | 27 ± 13 |
| **Cu** | 0 | 0.08 ± 0.15 |

**Table S6**. Seedling growth test, as the standard method required results obtained were computed from 2 parallel experiments (C_i_=20-100 mg/L, T=20 ^o^C, t=72 hours)

| **Sample (mg/L RBV-5R)** | **Lettuce seed** | | | **Mustard seed** | | |
| --- | --- | --- | --- | --- | --- | --- |
|  | **Root length (mm)** | **Number of germinated seeds** | **Root growth inhibition (%)** | **Root length (mm)** | **Number of germinated seeds** | **Root growth inhibition (%)** |
| 0 | 12.8 | 14.1 | - | 28.8 | 21.5 | - |
| 20 | 4.0 | 4.5 | 68.6 | 27.2 | 20.5 | 5.5 |
| 40 | 3.4 | 3.5 | 73.4 | 21.5 | 18.5 | 25.2 |
| 50 | 3.1 | 3.5 | 75.5 | 28.5 | 22.5 | 1.2 |
| 60 | 2.6 | 2 | 80 | 20.4 | 19 | 29.3 |
| 80 | 5.7 | 6 | 55.3 | 22.6 | 21 | 21.5 |
| 100 | 3.4 | 3.5 | 73.4 | 19.1 | 23 | 33.7 |

**
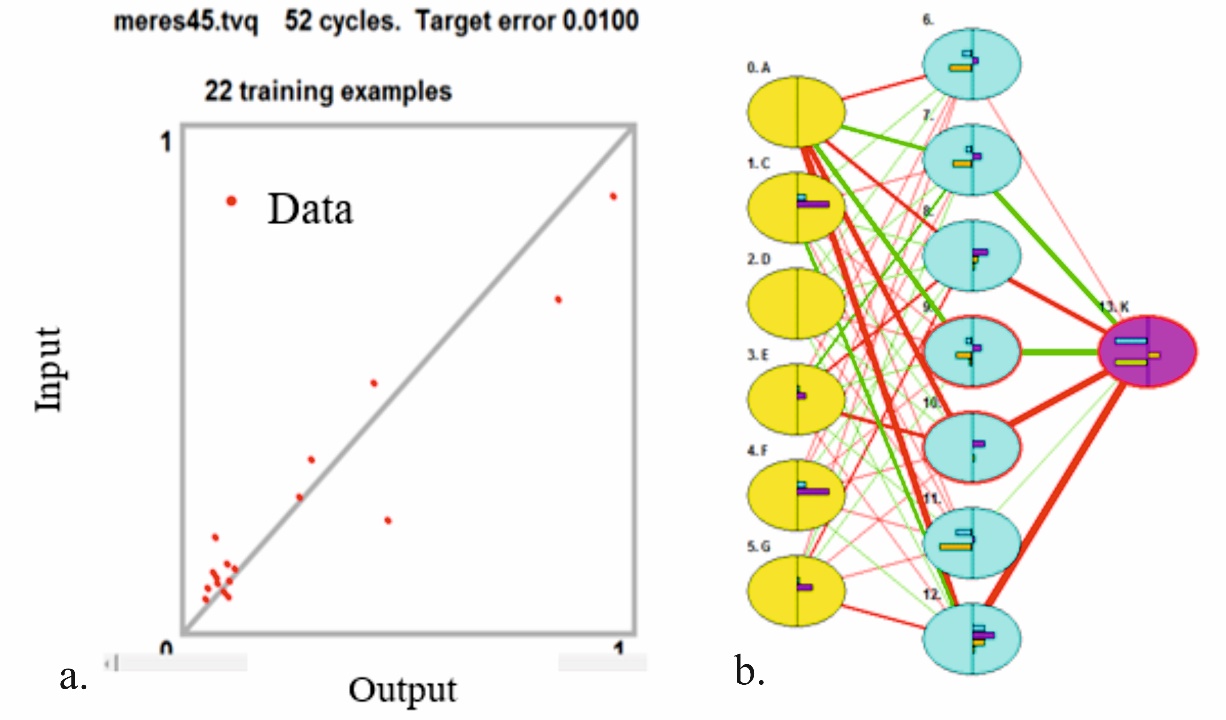
**

**Fig. S2**. Topology and performance of ANN.
